# Detection on Cell Cancer Using the Deep Transfer Learning and Histogram Based Image Focus Quality Assessment

**DOI:** 10.3390/s22187007

**Published:** 2022-09-16

**Authors:** Md Roman Bhuiyan, Junaidi Abdullah

**Affiliations:** Faculty of Computing and Informatics, Multimedia University, Persiaran Multimedia, Cyberjaya 63100, Selangor, Malaysia

**Keywords:** cancer detection, positive cell, negative cell, deep transfer learning

## Abstract

In recent years, the number of studies using whole-slide imaging (WSIs) of histopathology slides has expanded significantly. For the development and validation of artificial intelligence (AI) systems, glass slides from retrospective cohorts including patient follow-up data have been digitized. It has become crucial to determine that the quality of such resources meets the minimum requirements for the development of AI in the future. The need for automated quality control is one of the obstacles preventing the clinical implementation of digital pathology work processes. As a consequence of the inaccuracy of scanners in determining the focus of the image, the resulting visual blur can render the scanned slide useless. Moreover, when scanned at a resolution of 20× or higher, the resulting picture size of a scanned slide is often enormous. Therefore, for digital pathology to be clinically relevant, computational algorithms must be used to rapidly and reliably measure the picture’s focus quality and decide if an image requires re-scanning. We propose a metric for evaluating the quality of digital pathology images that uses a sum of even-derivative filter bases to generate a human visual-system-like kernel, which is described as the inverse of the lens’ point spread function. This kernel is then used for a digital pathology image to change high-frequency image data degraded by the scanner’s optics and assess the patch-level focus quality. Through several studies, we demonstrate that our technique correlates with ground-truth z-level data better than previous methods, and is computationally efficient. Using deep learning techniques, our suggested system is able to identify positive and negative cancer cells in images. We further expand our technique to create a local slide-level focus quality heatmap, which can be utilized for automated slide quality control, and we illustrate our method’s value in clinical scan quality control by comparing it to subjective slide quality ratings. The proposed method, GoogleNet, VGGNet, and ResNet had accuracy values of 98.5%, 94.5%, 94.00%, and 95.00% respectively.

## 1. Introduction

Cancer is a leading cause of mortality worldwide [1], and according to a study conducted by the American Cancer Society (ACS), roughly 600,920 Americans were predicted to die from cancer in 2017 [2]. Therefore, combating cancer is a significant problem for both researchers and clinicians [3]. Cancer diagnosis relies heavily on early detection, which may enhance the chances of long-term survival. Medical imaging is a crucial technology for the early identification and diagnosis of cancer. As is well known, medical imaging has been extensively used for early cancer diagnosis, monitoring, and follow-up after therapy [4]. Therefore, beginning in the early 1980s, computer-aided diagnosis (CAD) systems were developed to help physicians in evaluating medical pictures more efficiently [5]. For the identification and diagnosis of cancer, machine learning methods are commonly used in CAD systems, integrating medical imaging. The extraction of features is often a crucial step in the adoption of machine learning methods. Different approaches to feature extraction have been studied for various imaging modalities and cancer types [6,7,8,9,10,11,12,13,14,15,16,17,18,19,20,21]. For detecting mass areas in mammograms, bilateral image subtraction, difference of Gaussians, and Laplacian of Gaussian filters have been chosen as feature extractors [6,7,8,9,10] for breast cancer diagnosis. However, past work have mostly focused on generating appropriate feature descriptors in conjunction with machine learning algorithms for medical picture context learning. These approaches based on the extraction of features have several flaws. The shortcoming prevents the performance of CAD systems from being enhanced further. In recent years, rather than feature engineering, the relevance of representation learning has been stressed [22,23] in order to overcome the shortcomings and enhance the performance of CAD systems. Deep learning is a representation learning approach in which picture data are used to create hierarchical feature representations. Deep learning can be used to generate high-level representations of image features directly based on raw image data. In addition, with the support of massively parallel architectures and graphics processing units (GPUs), deep learning strategies have achieved significant success in a variety of fields in recent years, including picture identification, object detection, and speech recognition. Recent studies have indicated, for example, that CNNs [24] produce promising outcomes in cancer detection and diagnosis.

In this article, we present various prominent deep learning algorithms and proposed metrics for evaluating digital pathology images which uses some even derivative filter bases to generate human visual-system-like kernels.

The remainder of this paper is laid out as follows. Related work is discussed in Section 2. Then, in Section 3, we present the proposed method. In Section 4, we present the dataset preparation. In Section 5, we present the experimental results and a discussion. In Section 6, we present our conclusions.

## 2. Related Work

Cancer is the unregulated and abnormal division and development of cells inside the body. A brain tumor is a mass of abnormally dividing and proliferating brain tissue cells. Despite their rarity, brain tumors are among the most fatal types of cancer [25].

Brain tumors are classified as either primary or metastatic, depending on where they started. Although metastatic brain tumors originate in another organ and then spread to the brain, primary brain cancers arise directly from brain tissue. Gliomas are brain tumors that arise from glial cells. According to recent segmentation studies, they are the most common kind of brain tumor studied. Astrocytomas and oligodendrogliomas are examples of low-grade gliomas, but glioblastoma multiforme (GBM) is the most aggressive and prevalent kind of primary malignant brain tumor [26]. Gliomas are often treated with surgery, chemotherapy, and radiation therapy [27].

For better treatment options, early glioma detection is crucial. Imaging methods such as CT, SPECT, positron emission tomography (PET), magnetic resonance spectroscopy (MRS), and magnetic resonance imaging (MRI) may all help assess the size, location, and metabolism of brain tumors (MRI). Although both imaging techniques are used to provide the most thorough analysis of brain tumors, MRI is preferred due to its superior soft tissue contrast and general availability. Using radio frequency waves to stimulate target tissues and produce interior images under the influence of a strong magnetic field, MRI is a noninvasive in vivo imaging technology. During image capture, different MRI sequence images may be obtained by varying the excitation and repetition periods. The various tissue contrast images produced by these MRI modalities allow for the identification and segmentation of tumors and their subregions, as well as the provision of structural data. There are four primary methods for diagnosing a glioma using MRI: T1-weighted imaging, T2-weighted imaging, T1-Gd imaging, and fluid attenuated inversion recovery imaging (FLAIR). Approximately 150 slices of 2D images, depending on the equipment, are produced during MRI acquisition to represent the 3D brain volume. Additionally, the data become quite complex and perplexing when the slices of the necessary standard modalities are joined for diagnosis [28].

Although T2 images are used to characterize the edema region on the image, which produces a strong signal, T1 images are used to distinguish healthy tissues. The tumor border may be easily seen in T1-Gd images because of the high signal of the accumulated contrast agent (gadolinium ions) in the active cell zone of the tumor tissue. Necrotic cells do not interact with the contrast agent; therefore, they may be easily distinguished from the area of active cells in the tumor core based on the hypo-intense part of the tumor core in the same order. The reduced water molecule signals of the FLAIR images help to distinguish the edematous region from the cerebral fluid (CSF) [29].

Segmenting the tumor is necessary before beginning any therapy in order to protect healthy tissues, while damaging and getting rid of cancerous ones. Brain tumor segmentation comprises the diagnosis, delineation, and separation of tumor tissues from healthy brain tissues, including gray matter (GM), white matter (WM), and CSF. Tumor tissues include active cells, necrotic cores, and edema. In practical situations, this method still requires the manual annotation and segmentation of a number of multimodal MRI images. Since human segmentation requires a lot of time, the creation [29] of reliable automated segmentation algorithms to provide effective and objective segmentation has been a fascinating and popular research issue in recent years. Deep learning algorithms are now useful for segmentation, making them good candidates for this area of research [30]. In this article, we provide the foundations of the field of cancer diagnosis, including the procedures of cancer diagnosis, followed by the conventional classification strategies used by doctors, thus providing the reader with a historical perspective on cancer classification methodologies. The seven-point detection method, the Menzies method, and pattern analysis are provided as examples. The purpose of this bibliographic research was to provide academics interested in adopting deep learning and artificial neural networks for cancer diagnosis with an overview of the most current developments.

## 3. Proposed Method

**Figure 1:**
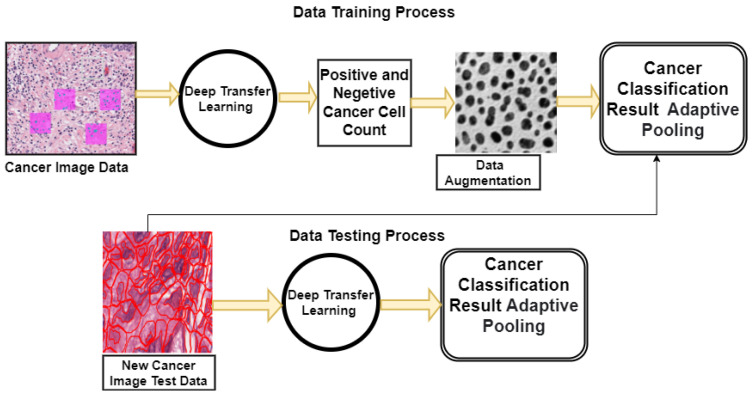
The proposed deep transfer learning model.

The FCNN-based architecture recommended for locating and categorizing malignant cells in cancer images is described in this Section. GoogLeNet, VGGNet, and ResNet—three well-known FCNN architectures—were used to independently extract different low-level aspects in the suggested system. For the purpose of the classification task, the aggregated attributes were added to a layer that was already completely linked, as shown in Figure 1. In the following subsections, we detail each phase of the suggested architecture.

### 3.1. Processes for Data Preprocessing and Augmentation

To eliminate the many forms of noise in tissue images, the preprocessing phase is crucial. The H&E-stained tissue micrographs are normalized in the suggested manner using the method described in [31]. A CNN requires vast data sets to improve its precision. The CNN’s performance is further hindered by the overfitting of small data sets. The network’s performance is low when applied to test data, but it excels when used on training data [32,33]. The data augmentation approach expands the sample size by using core image processing techniques and geometric alterations to picture data sets. In order to expand the image data set, several techniques such as color processing, transformation (including translation, scaling, and rotation), inversion, and noise perturbation have been used used.

### 3.2. Feature Extraction Using a Pre-Trained FCNN Model

Separate CNN architectures were originally implemented for feature extraction in classification tasks prior to their incorporation into a fully linked layer. Circularity, roundness, and compactness, as well as other shape descriptors, may be associated with these qualities. The feature extractors GoogLeNet [34], the Visual Geometry Group Network (VGGNet) [35], and Residual Networks (ResNet) [36] are used in the framework that is given for the classification of cancer in cytology images. These structures are pre-trained for a range of general image descriptors using the transfer learning theory [37], and then the important properties are recovered from microscopic images using that training. The following Sections cover the key components of each known FCNN architecture.

#### 3.2.1. GoogleNet

**Figure 2:**
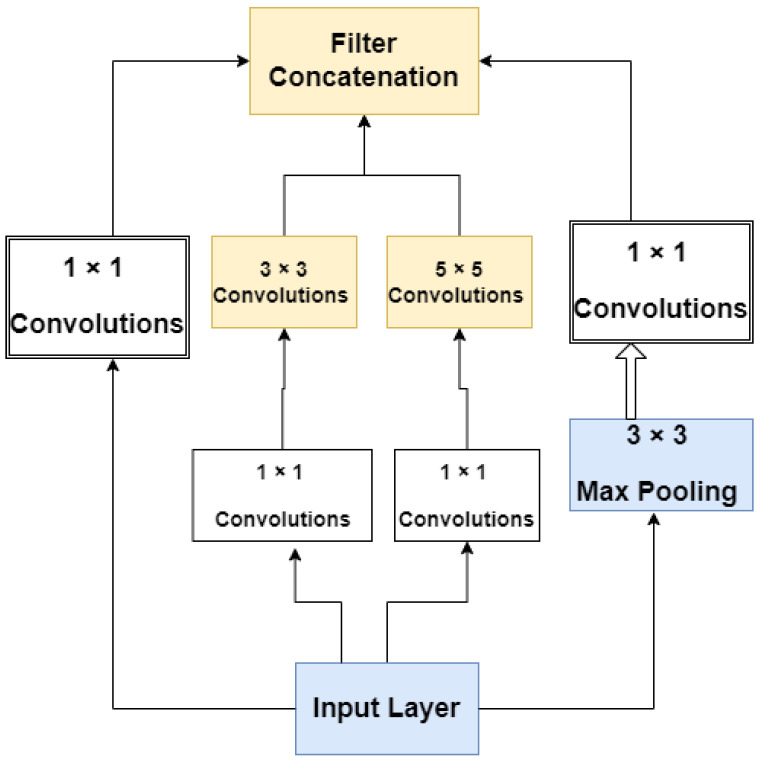
The model of GoogleNet’s structure [34].

This condensed network is made up of three layers of convolution, two levels of adaptive pooling, two layers of completely linked layers, and one layer that has its linear operations adjusted. We suggested a model that, using the architecture of GoogleNet, integrates numerous convolution filters of varied widths into a single new filter. As a result, both the number of perimeters and the computational cost are reduced. The essential organizational structure of GoogleNet is shown in Figure 2.

#### 3.2.2. Resnet

**Figure 3:**
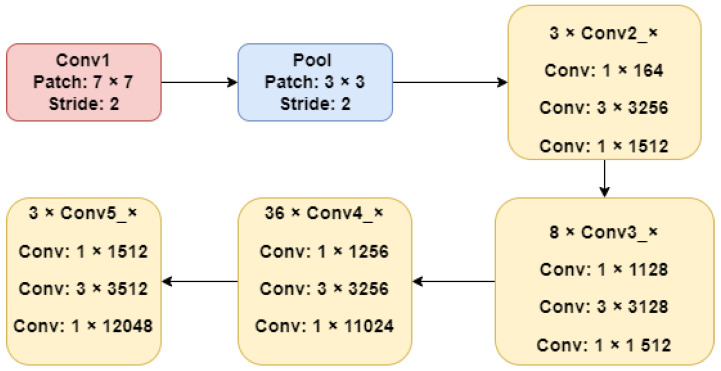
The model of the structure of ResNet [36].

In classification challenges related to ImageNet, ResNet, an extremely deep residual network, performs well [38]. Because of its deep structure, ResNet is able to minimize training time by combining convolution filters of various sizes. ResNet’s basic design is illustrated in Figure 3.

### 3.3. VGGNet

**Figure 4:**
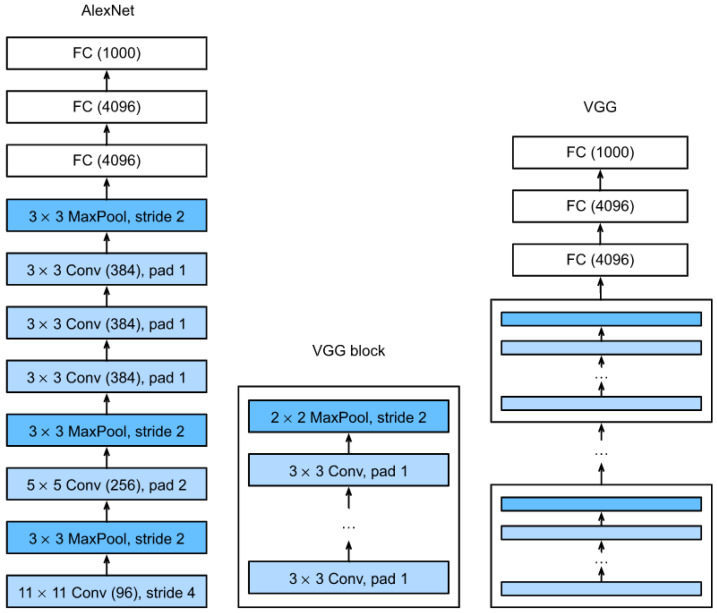
The model structure for VGGNet [35].

There are more convolution layers in VGGNet, which makes it comparable to AlexNet. VGGNet has 13 layers of convolution, rectification, and pooling, as well as three layers that are completely linked [35]. A 3 × 3 window filter and a 2 × 2 adaptive Pooling network are used in the convolution network. VGGNet outperforms AlexNet because of its simpler design [30]. Figure 4 illustrates VGGNet’s underlying structure.

### 3.4. Deep Transfer Learning

To train a CNN from scratch requires a massive amount of data, but it may be challenging to maintain a large data collection that covers all needed subjects. In the great majority of real-world applications, obtaining similar training and testing data is either impossible or very difficult. As a result, the notion of transfer learning has emerged. Transfer learning is one of the most well-known techniques in machine learning since it facilitates the transfer of previously acquired information to new situations with comparable features. In the first instance, the basic network is trained using the appropriate data set, and then it is applied to the desired task and trained using the desired data [37]. The selection of the pre-trained model, the difficulty level, and the similarity of the issues may be divided into two main transfer learning components. If the related issue is important in connection to the target problem, one can use a trained model. Target data sets that are smaller (less than 1000 pictures) and similar to the training data sets are more likely to suffer from overfitting (e.g., medical data sets, data sets of handwritten characters, data sets linked to cars, or data sets of biometrics). Similarly, if the amount of target data is equal to or greater than the amount of source data, the pre-trained model requires only modest alterations. The proposed system employs three CNN architectures (GoogleNet, VGGNet, and ResNet) to evaluate their transfer learning and fine-tuning capabilities. Transfer learning was applied in the ImageNet training of these three CNN architectures. This allowed the architecture to discover the underlying properties of several data sets without further training. Using average pooling, the fully connected layer classifies benign and cancerous cells by combining the quantity of data collected independently from each CNN design.

## 4. Dataset Preparation

### 4.1. Obtaining Pathology Images

For additional analysis, more than 138 high-resolution images of varying sizes (1600 × 1200, 1807 × 835, and 1807 × 896) were gathered and pre-classified. For each image, tissue samples were stained with May Grünwald-Giemsa (MGG) and hematoxylin and eosin (H&E) stains. The amount of images in each class varied, with 58 in the advanced class and only 20 in the normal class. Because of the reduced resolution of the input, the images were pre-processed into tiny, non-overlapping patches in order to better capture the cell properties necessary for calculating their grade.

### 4.2. Image Dataset

The slicing of the 138 original shots yielded 6468 patches, representing a 357% increase in the amount of images. In all, 55% (4358) of the background and non-tissue information patches were eliminated. May Grünwald-Giemsa (MGG) and hematoxylin and eosin (H&E) stain pathology images use a merged dataset of all MGG and H&E stained images. Hematoxylin and eosin (H&E) stain is the most commonly used stain for light microscopy in histopathology laboratories due to its relative simplicity of use and the ability to disclose a wide range of normal and diseased cell and tissue components. Five patients diagnosed with malaria infections had their peripheral blood (PB) smears stained with May Grünwald-Giemsa (MGG) every day at the Core Laboratory at the Hospital Clinic de Barcelona. Using an Olympus B × 43 microscope with 1000 × magnification and a digital camera, digital images were obtained (Olympus DP73). The images included inside the collection were JPG files (RGB, 2400 × 1800 pixels).

### 4.3. Cross-Validation and Training-validation

To evaluate the DL model, 80% and 20% images from each dataset were sorted into the training and validation sets, respectively. Using a K = 5 K-fold cross-validation approach, we divided the MGG, H&E, and mixed datasets into five equal sections and used them to generate five new cross-validation sets including five new copies of each dataset (e.g., MGG Set 1 to MGG Set 5 for MGG). Eighty percent of the images used for training and validation in each batch were distinct (20%). The average of the five training cycles was utilized to measure improvements.

## 5. Experimental Results and Discussion

### 5.1. Cancer Cell Detection Result

**Figure 5:**
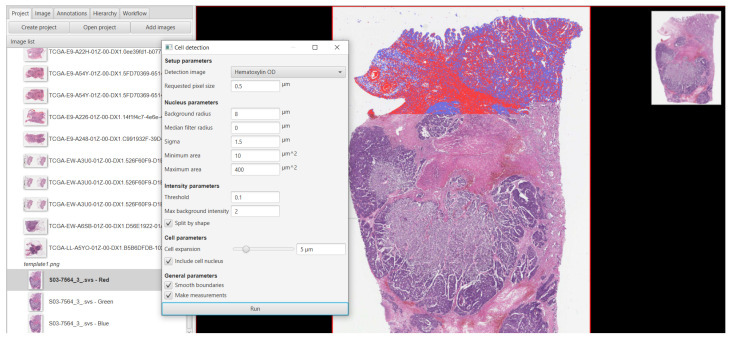
Cancer cell detection process.

**Figure 6:**
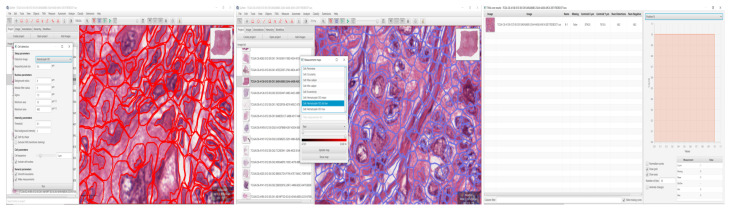
A typical approach used for TMA analysis has the following characteristics (from left to right): the development of a multi-slide project that includes automated TMA dearraying, stain estimation, cell identification and feature calculation, trainable cell classification, batch processing, and survival analysis.

In this stage of the study, we tried to detect the cells based on the cancer images. For this experiment, we used 100 cancer images and we achieved an average detection of 70% of cells. At the same time, we tried to find the positive and negative cell detection results from the cancer images. For the positive and negative cell detection tasks, we obtained accuracy values of 85% and 80%, respectively. Figure 5 shows the positive and negative cell detection results and Figure 6 shows the TMA analysis.

### 5.2. Proposed Method Results

The proposed system uses transfer learning to take the knowledge gained from training on three distinct FCNN architectures (GoogLeNet, VGGNet, and ResNet) and apply it to the task of extracting features jointly. An array of modern methods and a set of integrated characteristics are used to evaluate the single CNN’s output.

**Figure 7:**
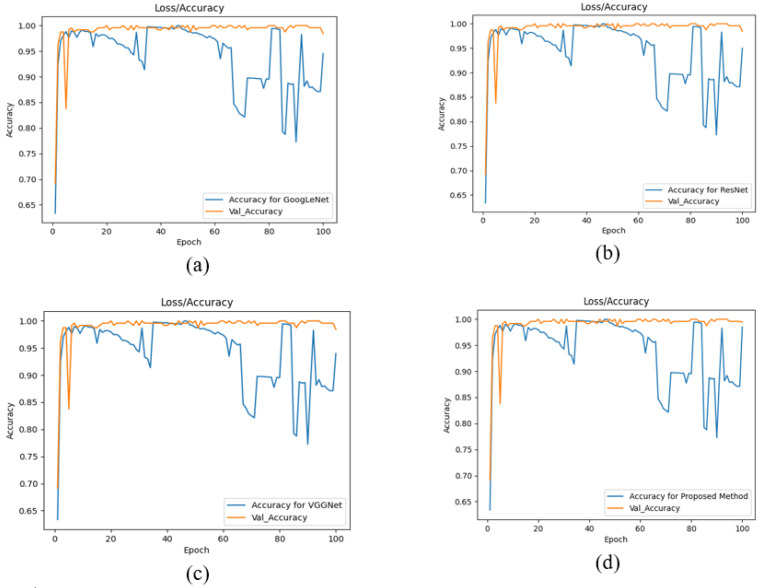
Analysis results with graphical representations—(**a**) GoogLeNet (**b**) VGGNet (**c**) ResNet, and (**d**) the proposed method.

Furthermore, during the testing of the proposed technique, the data were separated into training and testing data sets. According to the 80%/20% split, 80% of the data were expected to be used to train CNN models, whereas the remaining 20% were likely to be used to test the CNN models. The proposed approach based on data splittingwas compared to those of the various CNN architectures described in Table 1. Under “Class Name,” Table 1 displays the class of cancer (B—benign or M—malignant) and its accompanying precision, recall, F1 score, and accuracy. It also provides the average accuracy of each design based on splitting processes. When compared to individual structures, the suggested framework delivered more accurate images of cancer cells. Figure 7 shows a graphical representation of the results.

### 5.3. Accuracy Comparison with Different Approaches

As shown in Table 2, we compared the proposed framework’s outcomes to four other well-known methodologies to observe the performance of the suggested design. Table 2 showed that the approaches in [39,40,41,42] were accurate to 92.63%, 90.0%, 97.0%, and 97.5%, respectively, whereas the findings produced using the suggested framework were accurate to 98.50%, which was greater than the values of all four other methods. These findings demonstrate the approach’s superior accuracy when compared to other approaches of a similar kind.

## 6. Conclusions

To better identify and classify cancerous tumors, we developed a new deep learning system based on transfer learning. This method uses three CNN architectures (GoogleNet, VGGNet, and ResNet) to extract features from tumor cytology images and then combines them to enhance classification accuracy. A similar idea, data augmentation, was introduced by us to expand the size of a data set to enhance a CNN structure’s performance. Finally, the proposed framework’s performance was compared to several CNN designs, as well as existing approaches. Without having to start from scratch, the suggested framework produced outstanding accuracy results, which could boost classification efficiency. Handcrafted and CNN characteristics will be employed together in the future to boost classification accuracy even more. The suggested experimental findings revealed that the performance measures of accuracy, precision, recall, and F1 scores were 98.5%, 96.0%, 97.0%, and 93.0%, respectively. As the major focus of this work was deep learning for cancer diagnosis, the most important topics to describe to our readers are all possible deep learning diagnostic procedures.

## Figures and Tables

**Table 1:** Using data splitting, we compared the proposed framework to several CNN architectures.

Model	Data Splintting	Class Name	Precision	Recall	F1 Score	Accuracy
GoogLeNet	80–20%	B M	95.0	96.0	93.0	94.5%
VGGNet	80–20%	B M	91.0	96.0	93.0	94.00%
ResNet	80–20%	B M	95.0	97.0	90.0	95.00%
Proposed Method	80–20%	B M	96.0	97.0	93.0	98.5%

**Table 2:** Accuracy comparisons with other models.

Model	Accuracy
Nguyen [39]	92.63%
Awan [40]	90.00%
Kensert [41]	97.00%
Vesal [42]	97.50%
Proposed method	98.50%

## Data Availability

Herewith, we have attached the dataset link, http://histoqcrepo.com/ (accessed on 21 September 2021).

## References

[1] Torre L.A., Bray F., Siegel R.L., Ferlay J., Lortet–Tieulent J., Jemal A. (2015). Global cancer statistics, 2012. CA Cancer J. Clin..

[2] American Cancer Society (2017). Cancer Facts & Figures 2017.

[3] Siegel R.L., Miller K.D., Jemal A. (2016). Cancer statistics, 2016. CA Cancer J. Clin..

[4] Fass L. (2008). Imaging and cancer: A review. Mol. Oncol..

[5] Doi K. (2007). Computer-aided diagnosis in medical imaging: Historical review, current status and future potential. Comput. Med. Imaging Graph..

[6] Yin F.F., Giger M.L., Vyborny C.J., Schmidt R.A. (1994). Computerized detection of masses in digital mammograms: Automated alignment of breast images and its effect on bilateral-subtraction technique. Med. Phys..

[7] Beller M., Stotzka R., Müller T., Gemmeke H. (2005). An example-based system to support the segmentation of stellate lesions. Bildverarb. Med..

[8] te Brake G.M., Karssemeijer N., Hendriks J.H. (2000). An automatic method to discriminate malignant masses from normal tissue in digital mammograms. Phys. Med. Biol..

[9] Eltonsy N.H., Tourassi G.D., Elmaghraby A.S. (2007). A concentric morphology model for the detection of masses in mammography. IEEE Trans. Med. Imaging.

[10] Wei J., Sahiner B., Hadjiiski L.M., Chan H.P., Petrick N., Helvie M.A., Roubidoux M.A., Ge J., Zhou C. (2005). Computer-aided detection of breast masses on full field digital mammograms. Med. Phys..

[11] Hawkins S.H., Korecki J.N., Balagurunathan Y., Gu Y., Kumar V., Basu S., Hall L.O., Goldgof D.B., Gatenby R.A., Gillies R.J. (2014). Predicting outcomes of nonsmall cell lung cancer using CT image features. IEEE Access.

[12] Aerts H.J., Velazquez E.R., Leijenaar R.T., Parmar C., Grossmann P., Cavalho S., Bussink J., Monshouwer R., Haibe-Kains B., Rietveld D. (2014). Decoding tumour phenotype by noninvasive imaging using a quantitative radiomics approach. Nat. Commun..

[13] Balagurunathan Y., Gu Y., Wang H., Kumar V., Grove O., Hawkins S., Kim J., Goldgof D.B., Hall L.O., Gatenby R.A. (2014). Reproducibility and prognosis of quantitative features extracted from CT images. Transl. Oncol..

[14] Barata C., Marques J.S., Celebi M.E. Improving dermoscopy image analysis using color constancy. Proceedings of the 2014 IEEE International Conference on Image Processing (ICIP).

[15] Barata C., Marques J.S., Rozeira J. (2012). A system for the detection of pigment network in dermoscopy images using directional filters. IEEE Trans. Biomed. Eng..

[16] Barata C., Ruela M., Mendonça T., Marques J.S. (2014). A bag-of-features approach for the classification of melanomas in dermoscopy images: The role of color and texture descriptors. Computer Vision Techniques for the Diagnosis of Skin Cancer.

[17] Sadeghi M., Lee T.K., McLean D., Lui H., Atkins M.S. (2013). Detection and analysis of irregular streaks in dermoscopic images of skin lesions. IEEE Trans. Med. Imaging.

[18] Zikic D., Glocker B., Konukoglu E., Criminisi A., Demiralp C., Shotton J., Thomas O.M., Das T., Jena R., Price S.J. Decision forests for tissue-specific segmentation of high-grade gliomas in multi-channel MR. Proceedings of the International Conference on Medical Image Computing and Computer—Assisted Intervention.

[19] Meier R., Bauer S., Slotboom J., Wiest R., Reyes M. (2013). A Hybrid Model for Multimodal Brain Tumor Segmentation.

[20] Pinto A., Pereira S., Correia H., Oliveira J., Rasteiro D.M., Silva C.A. Brain tumour segmentation based on extremely randomized forest with highlevel features. Proceedings of the 37th Annual International Conference on IEEE Engineering in Medicine and Biology Society (EMBC, 2015).

[21] Tustison N.J., Shrinidhi K., Wintermark M., Durst C.R., Kandel B.M., Gee J.C., Grossman M.C., Avants B.B. (2015). Optimal symmetric multimodal templates and concatenated random forests for supervised brain tumor segmentation (simplified) with ANTsR. Neuroinformatics.

[22] Bengio Y., Courville A., Vincent P. (2013). Representation learning: A review and new perspectives. IEEE Trans. Pattern Anal. Mach. Intell..

[23] LeCun Y., Bengio Y., Hinton G. (2015). Deep learning. Nature.

[24] Xu B., Wang N., Chen T., Li M. (2015). Empirical evaluation of rectified activations in convolutional network. arXiv.

[25] De Angelis L.M. (2001). Brain Tumors. N. Engl. J. Med..

[26] Gliomas D.A. (2009). Recent Results in Cancer Research.

[27] Stupp R. (2007). Malignant glioma: ESMO clinical recommendations for diagnosis, treatment and follow-up. Ann. Oncol..

[28] Menze B.H., Jakab A., Bauer S., Kalpathy-Cramer J., Farahani K., Kirby J., Burren Y., Porz N., Slotboom J., Wiest R. (2015). The Multimodal brain tumor image segmentation benchmark (brats). IEEE Trans. Med. Imaging.

[29] Drevelegas A., Papanikolou N. (2011). Imaging Modalities in Brain Tumors Imaging of Brain Tumors with Histological Correlations.

[30] Ahmad S., Ullah T., Ahmad I., Al-Sharabi A., Ullah K., Khan R.A., Rasheed S., Ullah I., Uddin M., Ali M. (2022). A Novel Hybrid Deep Learning Model for Metastatic Cancer Detection. Comput. Intell. Neurosci..

[31] Macenko M., Niethammer M., Marron J.S., Borland D., Woosley J.T., Guan X., Schmitt C., Thomas N.E. A method for normalizing histology slides for quantitative analysis. Proceedings of the 2009 IEEE International Symposium on Biomedical Imaging: From Nano to Macro.

[32] Krizhevsky A., Sutskever I., Hinton G.E. Imagenet classification with deep convolutional neural networks. Proceedings of the Advances in Neural Information Processing Systems.

[33] Cireşan D., Meier U., Schmidhuber J. (2012). Multicolumn deep neural networks for image classification. arXiv.

[34] Szegedy C., Liu W., Jia Y., Sermanet P., Reed S., Anguelov D., Erhan D., Van-Houcke V., Rabinovich A. Going deeper with convolutions. Proceedings of the IEEE Conference on Computer Vision and Pattern Recognition.

[35] Simonyan K., Zisserman A. (2014). Very deep convolutional networks for large-scale image recognition. arXiv.

[36] He K., Zhang X., Ren S., Sun J. Deep residual learning for image recognition. Proceedings of the IEEE Conference on Computer Vision and Pattern Recognition.

[37] Yosinski J., Clune J., Bengio Y., Lipson H. How transferable are features in deep neural networks?. Proceedings of the Advances in Neural Information Processing Systems.

[38] Yu Y., Lin H., Yu Q., Meng J., Zhao Z., Li Y., Zuo L. (2015). Modality classification for medical images using multiple deep convolutional neural networks. J. Comput. Inf. Syst..

[39] Nguyen L.D., Lin D., Lin Z., Cao J. Deep cnns for microscopic image classification by exploiting transfer learning and feature concatenation. Proceedings of the 2018 IEEE International Symposium on Circuits and Systems (ISCAS).

[40] Awan R., Koohbanani N.A., Shaban M., Lisowska A., Rajpoot N. Context-aware learning using transferable features for classification of breast cancer histology images. Proceedings of the International Conference Image Analysis and Recognition.

[41] Kensert A., Harrison P.J., Spjuth O. (2019). Transfer learning with deep convolutional neural networks for classifying cellular morphological changes. SLAS Discov. Adv. Life Sci. R&D.

[42] Vesal S., Ravikumar N., Davari A., Ellmann S., Maier A. Classification of breast cancer histology images using transfer learning. Proceedings of the International Conference Image Analysis and Recognition.

